# Comparison of the Seven Interleukin-32 Isoforms’ Biological Activities: IL-32θ Possesses the Most Dominant Biological Activity

**DOI:** 10.3389/fimmu.2022.837588

**Published:** 2022-02-25

**Authors:** Saerok Shim, Siyoung Lee, Yasmin Hisham, Sinae Kim, Tam T. Nguyen, Afeisha S. Taitt, Jihyeong Hwang, Hyunjhung Jhun, Ho-Young Park, Youngmin Lee, Su Cheong Yeom, Sang-Yeob Kim, Yong-Gil Kim, Soohyun Kim

**Affiliations:** ^1^ Laboratory of Cytokine Immunology, Department of Biomedical Science and Technology, Konkuk University, Seoul, South Korea; ^2^ College of Veterinary Medicine, Konkuk University, Seoul, South Korea; ^3^ Technical Assistance Center, Korea Food Research Institute, Wanju, South Korea; ^4^ Research Group of Functional Food Materials, Korea Food Research Institute, Wanju, South Korea; ^5^ Department of Medicine, Pusan Paik Hospital, Collage of Medicine, Inje University, Busan, South Korea; ^6^ Graduate School of International Agricultural Technology, Seoul National University, Pyeongchang, South Korea; ^7^ Convergence Medicine Research Center, Asan Institute for Life Science, Asan Medical Center, Seoul, South Korea; ^8^ Division of Rheumatology, Department of Internal Medicine, Asan Medical Center, University of Ulsan College of Medicine, Seoul, South Korea

**Keywords:** interleukin-32, recombinant protein, isoforms, IL-32θ, inflammatory cytokine

## Abstract

Cytokines are significantly associated with the homeostasis of immune responses in health and disease. Interleukin-32 (IL-32) is a cytokine originally discovered in natural killer cell transcript 4. IL-32 with different disorders has been described in terms of pathogenesis and the progression of diseases. Clinical studies have investigated IL-32 under various conditions, such as viral infection, autoimmune diseases, inflammatory diseases, certain types of cancer, vascular disease, and pulmonary diseases. The high expression of IL-32 was identified in different tissues with various diseases and found to have multiple transcripts of up to seven isoforms. However, the purification and biological activities of these isoforms have not been investigated yet. Therefore, in this study, we purified and compared the biological activity of recombinant IL-32 (rIL-32) isoforms. This is the first time for seven rIL-32 isoforms (α, β, δ, γ, ϵ, ζ, and θ) to be cloned and purified using an *Escherichia coli* expression system. Next, we evaluate the biological activities of these seven rIL-32 isoforms, which were used to treat different types of cells by assessing the levels of inflammatory cytokine production. The results revealed that rIL-32θ possessed the most dominant biological activity in both immune and non-immune cells.

## Introduction

Interleukin-32 (IL-32) cytokine was cloned in 1992 from natural killer cells and was formerly named natural killer cell transcript 4 (NK4). NK4 was renamed IL-32 in 2005 because it has a cytokine property (1, 2). It was found to induce several inflammatory cytokines, such as tumor necrosis factor-α (TNFα), interleukin-6 (IL-6), macrophage inflammatory protein-2 (MIP2), interleukin-8 (IL-8), and interleukin-1 beta (IL-1β), and IL-32 acts like a proinflammatory cytokine (1–3).

Nevertheless, since its discovery, much knowledge remains to be determined. For the most part, its specific surface receptor has yet to be defined. Proteinase 3 (PR3) binds to IL-32 with very high affinity (4). PR3 is a serine proteinase produced from neutrophils as an enzyme, whereas it is also expressed on the membrane of monocytes. The possibility of IL-32 binding to integrins has been suggested (5), and this result was based on its amino acid composition containing an RGD motif, which ubiquitously presents in various genes. The IL-32 amino acid sequence has no known cytokine homolog; in addition, IL-32 was detected in most mammals except rodents (6).

A previous study reported that the IL-32 gene is composed of eight exons and presents within human chromosome 16p13.3 (1). According to its alternative splicing sites, more than seven transcripts have been suggested. However, seven isoforms with nine exons were described to be translated from its messenger RNA transcript (7). These isoforms are IL-32α, IL-32β, IL-32γ, IL-32δ, IL-32ε, IL-32ζ, and IL-32θ. As each isoform was discovered separately, the cell type, condition, and isoform function were varied. IL-32α, IL-32β, IL-32γ, and IL-32δ were mainly identified in IL-2-stimulated human NK cells; on the other hand, IL-32ε and IL-32ζ were found to be expressed in activated T cells (8). Lastly, IL-32θ was discovered from dendritic cells and Jurkat cells of human leukemia T cell line (9). These isoforms exhibited distinct effects in different conditions. Among the seven IL-32 isoforms, IL-32γ is the most-studied isoform, which also has the longest amino acid sequence.

IL-32 plays a vital modulator role in the pathogenesis of different diseases. Its involvement has been reported in various cancers, infections, and autoimmune and inflammatory disorders (6, 10, 11). Most autoimmune and inflammatory diseases associated with IL-32 are rheumatoid arthritis (RA), inflammatory bowel disease (IBD), psoriasis, chronic obstructive pulmonary disease (COPD), and asthma (3, 7, 10, 12–14). However, these clinical studies determined the levels of circulating IL-32 and then compared the patients to healthy controls. These studies fail to characterize the differences in IL-32 isoforms. However, the protein identification of each IL-32 isoform is subjected to a significant limitation because of the lack of a specific antibody to detect the IL-32 variant. Moreover, IL-32 secreted proteins are not easily purified since the structures of IL-32 isoforms are not thoroughly appraised.

At present, we were able to purify seven rIL-32 isoforms: with IL-32α, -β, -δ, and -γ purified in our previous study, whereas IL-32ϵ, -ζ, and -θ were puried for the first time in this study. Next, we assessed the biological activities of the seven rIL-32 isoforms in various cells by examining the production of inflammatory cytokines, such as IL-6, IL-8, TNFα, and MIP2. Seven rIL-32 isoforms show a different biological activity regarding different cell types.

## Materials and Methods

### Isoform Cloning and Expression

**Figure 1:**
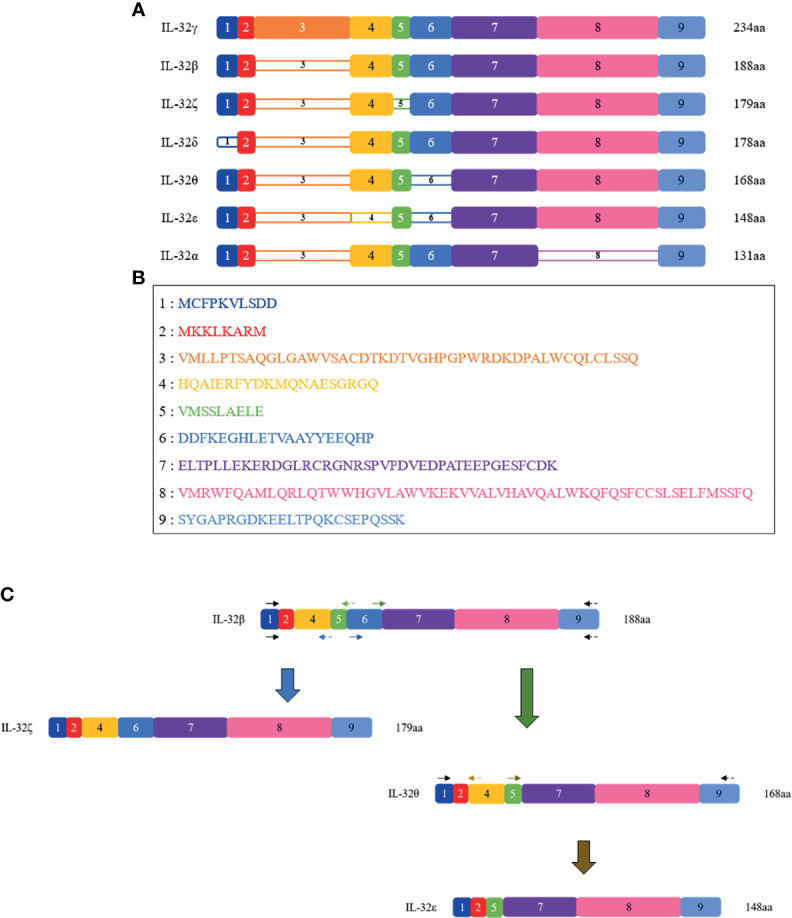
Schematic representations of the seven IL-32 isoforms. **(A)** Seven isoforms of IL-32 are shown with their present/absent domains. The name of each isoform is shown on the left, and their length in amino acid (aa) is on the right. The colored, numbered box represents exons from 1 to 9, numbered in line with the order of N-terminal; dense-colored boxes are indicated for the existing exons and decolored boxes for the absent exon. Starting from the longest isoform, IL-32γ isoform is represented on the top as it is the longest variant with 234 amino acid residues and the only one that contains all 9 exons, while on the bottom the shortest variant is present, which is IL-32α isoform with 131 amino acid residues. **(B)** Amino acid sequence of each exon. **(C)** Schematic PCR-based construction of IL-32ζ, IL-32θ, and IL-32ϵ; using IL-32β as a template, IL-32ζ and θ were built. Next, using IL-32θ as a template, IL-32ϵ was built. The black arrows represent outer primers and are the same in all constructs; the blue, green, and gold arrows represent the inner primers used to build IL-32ζ, IL-32θ, and IL-32ϵ, respectively. The forward primers are indicated as solid arrows, while the reverse primers are indicated as dashed arrows.

All seven isoforms were cloned into pPROEX/HTa from Takara (Shiga, Japan) as previously described (8). IL-32α, -β, -γ, and -δ isoforms were cloned earlier, and the remaining three IL-32ϵ, -ζ, and -θ isoforms were constructed in this study for the first time using the closest isoform as the template, as shown in **Figure 1**. Briefly, pPROEX/HTa IL-32β plasmid was used to construct IL-32ζ and -θ. Then, IL-32θ was used to construct IL-32ϵ (**Figure 1C**). For the construction of a new isoform plasmid vector, we used overlap extension PCR with primers as indicated in **Figure 1C**. All PCR products were designed to have EcoRI and XbaI restriction enzyme sites in their 5′ and 3′ ends. Next, the PCR products were ligated into an expression vector using EcoRI and XbaI restriction enzymes (Takara) and confirmed by DNA sequencing analysis in Cosmogen (Seoul, Korea). These expression vectors were transformed into BL21-Codon Plus from Stratagene (San Diego, CA, USA) by heat shock method.

### Recombinant Protein Expression and Purification

Seven recombinant rIL-32 (α, -β, -γ, -δ, -ϵ, -ζ, and -θ) proteins were expressed in *E. coli* with 4-h isopropyl β-D-1-thiogalactopyranoside induction at 37°C. rIL-32β, -γ, and -θ were purified with Ni-NTA agarose from Qiagen (Hilden, Germany), and the others were purified with TALON^®^ Magnetic Bead (Takara) using his^6^-tag at the N-terminus of rIL-32 isoform proteins. Among the affinity-purified proteins, rIL-32β, -γ, and -ϵ were subjected to a high-performance liquid chromatography column from Grace (Stockbridge, GA), and rIL-32α, -δ, -ζ, and -θ were subjected to an anion exchange column (HiTrap Q FF, 1 ml) from GE Healthcare (Chicago, IL, USA). After that, we checked their concentration by silver staining, Bradford assay, and BCA assay. Next, to check the bands of purified rIL-32 isoform proteins, we did western blotting with mouse anti-his^6^-tag mAb from R&D system (Minneapolis, MN, USA). The rIL-32 proteins were tested with a LAL chromogenic endotoxin quantitation kit from Thermo Fisher (Waltham, MA, USA). The endotoxin level was below 0.5 EU per 1 μg of rIL-32 protein, which is approximately 0.05 ng in 1 μg of rIL-32.

### Gene Expression Analysis

The expression levels of IL-32 in normal tissues were identified using GTEx Portal (https://www.gtexportal.org/home/).

### Cell Culture and Cytokine Assays

THP-1 and Raw 264.7, A459 cell lines were obtained from ATCC (Manassas, VA, USA). The THP-1 monocytes and Raw 264.7 cells were cultured in RPMI 1640 medium supplemented with 10% fetal bovine serum (FBS), 100 μg/ml penicillin, and 100 μg/ml streptomycin. A549 was cultured in Ham’s F12K medium containing the same reagents. Mouse embryonic fibroblasts (MEFs) were cultured in Dulbecco’s modified Eagle’s medium (DMEM) medium containing the same reagents. All cell culture media were from Welgene Biotech (Taipei, Taiwan). The culture condition was as follows: under humidified 5% CO_2_ at 37°C. THP-1 (2.5 × 10^4^/well), Raw 264.7 (5.0 × 10^4^/well), and A549 (2.5 × 10^4^/well) were seeded in a 96-well plate of 100 μl volume. THP-1 and Raw 264.7 were treated with different concentrations of purified rIL-32 isoforms (11.1, 33.3, and 100 ng/ml) in 100-μl-volume media, and the control was treated with the media alone. A549 and MEFs were treated with different concentrations of purified rIL-32 isoforms (100, 200, and 1,000 ng/ml) in 100-μl-volume media, and the control was treated with media alone. After 18 h of stimulation, the supernatants of THP-1 and Raw 264.7 were assessed for human IL-8 and mouse TNFα measurements, respectively. The A549 and MEF supernatants were assessed for human and mouse IL-6 measurements, respectively. All tested cytokines were determined by ELISA kits (R&D system).

### Cell Isolation From Mouse and Cytokine Assays

To verify that rIL-32 isoforms induced various cytokines in primary cells, we prepared splenocytes, bone marrow cells, and lung cells from C57BL/6 from Orient Bio (Seoul, Korea). All animal procedures were reviewed and approved by the Konkuk University Institutional Animal Care Committee. A C57BL/6 mouse was dissected, and the spleen, bone, and lung were isolated. We mashed the spleens and collected bone marrow cells from bones. These were centrifugated, washed with Dulbecco’s phosphate-buffered saline, and suspended in RPMI1640 medium supplemented with 10% FBS, 100 μg/ml penicillin, and 100 μg/ml streptomycin. In the case of lung cells, these were chopped, centrifugated, treated with collagenase V, and suspended in RPMI 1640 medium supplemented with 10% FBS, 100 μg/ml penicillin, and 100 μg/ml streptomycin. The MEF cells were prepared as follows: the fetus was isolated at 13.5 days of pregnancy. The fetus was chopped and digested with trypsin and DNase 1 and then suspended and cultured in DMEM medium supplemented with 10% FBS, 100 μg/ml penicillin, and 100 μg/ml streptomycin. The isolated primary mouse cells were seeded as follows: splenocytes (4.0 × 10^6^/well), bone marrow cells (1.0 × 10^6^/well), lung cells (2.5 × 10^4^/well), and MEFs (2.5 × 10^4^/well). After having been stimulated for 18 h with rIL-32 isoforms, mouse TNFα, mouse IL-6, and mouse MIP2 were assessed by using the ELISA set (R&D system).

### Statistical Analysis

All data were analyzed by Graph Pad Prism v.9 to perform one-way or two-way ANOVA, followed by Tukey’s *post-hoc* analysis. *P*-values <0.05 were considered statistically significant and were indicated in the figure legends.

## Results

### The Construction, Expression, and Purification of Seven rIL-32 Isoforms

**Figure 2:**
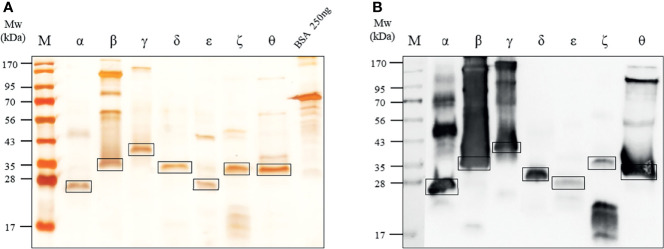
Expression of seven rIL-32 isoform proteins. **(A)** 10% SDS-PAGE analysis followed by silver staining for the seven purified rIL-32 isoforms of rIL-32 after a multi-step purification procedure compared with a known concentration of bovine serum albumin. The molecular weight (kDa) and rIL-32 isoforms are indicated at the top of their respective lanes in the following arrangement: α, β, γ, δ, ϵ, ζ, and θ. kDa; kilodalton. **(B)** Western blot analysis for seven rIL-32 proteins were loaded and probed with mouse anti-his^6^ tag mAb.

Seven IL-32 isoforms were constructed and cloned as shown in **Figure 1A**. IL-32 was divided into 9 small domains, and domain 8 is the longest. The amino acid sequence of each domain was illustrated with different colors, as shown in **Figure 1B**, corresponding to the color of the domain in **Figure 1A**. Each isoform of complete open reading frame in pPROEX/HTa *E. coli* had its expression vector confirmed by DNA sequencing. Multi-step (his^6^-tag purification and ion-exchange chromatography or high-performance liquid chromatography) purification was employed to obtain seven pure isoforms of rIL-32 protein. **Figure 2A** shows the 10% SDS-PAGE analysis of rIL-32 isoforms, with the dominant bands of each isoform corresponding to its theoretical molecular size as follows: IL-32α: 19.8 kDa, IL-32β: 25.5 kDa, IL-32γ: 31.5 kDa, IL-32δ: 24.2 kDa, IL-32ϵ: 20.7 kDa, IL-32ζ: 24.4 kDa, and IL-32θ: 23.0 kDa, plus 5.4 kDa of N-terminus his^6^-tag. All seven rIL-32 isoforms were migrated slowly; therefore, the molecular weight in silver staining was slightly higher than the actual molecular weight of each rIL-32 isoform. In addition to this, some rIL-32 isoforms appeared as a dimer and multiple bands. To confirm whether these bands were purified rIL-32 from *E. coli* or not, we did a western blot analysis using mouse anti-his^6^ tag mAb. As shown in **Figure 2B**, all protein bands in silver staining were found to be bound with mouse anti-his^6^ tag mAb to confirm the purity of the final seven rIL-32 isoform proteins.

### Gene Expression of IL-32 in Different Cell Types (Using GTEx)

**Figure 3:**
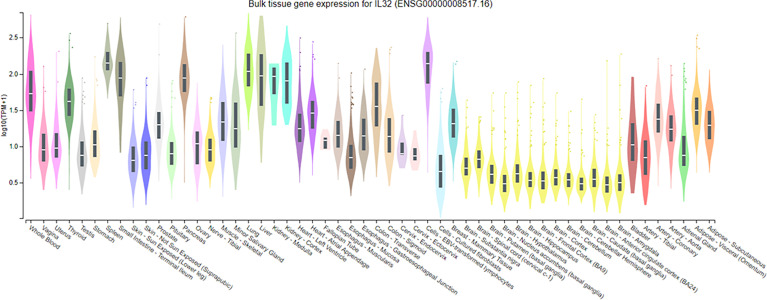
Expression of IL-32 in normal tissue samples. IL-32 gene expression analysis on normal tissue using GTEx portal; values of expression are shown in transcript per million and are calculated from a gene model with the isoforms collapsed to a single gene. The box plots are shown as median and 25th and 75th percentiles; the outliers are defined as above or below 1.5 times the interquartile range and are shown as points. The highest IL-32 expression was found in the spleen, followed by Epstein–Barr virus-transformed lymphocytes, and lung, whereas the lowest was observed in all tissue types of the brain.

It has been reported that the expression of IL-32 cytokine is increased in a variety of inflammatory autoimmune diseases and certain infections and cancers. We evaluated the expression of IL-32 in normal tissues using GTEx portal. Additionally, IL-32 is expressed in many cell types, including immune and non-immune cells, exhibiting different activities, which may be due to differences in cell types and/or stimulus and different isoform expressions related to cell types. However, a comparison of the activity of IL-32 isoforms has not been elucidated. IL-32 expression in normal tissues revealed that the highest expression of IL-32 was found in the spleen, followed by Epstein–Barr virus-transformed lymphocytes and then lung tissues (**Figure 3**). Therefore, we compared the activity of the seven IL-32 isoforms in several cell types, including immune cells, lung cells, and fibroblasts. THP-1 (human-derived monocytes) and Raw 264.7 (mouse-derived monocytes/macrophages) cell lines were used to evaluate the biological activity of rIL-32 isoforms. Primary mouse bone marrow and splenocytes were also isolated to evaluate the biological activity of rIL-32 isoforms. rIL-32 promotes the differentiation of monocytes into macrophage-like cells, inducing proinflammatory cytokines such as TNFα, IL-6, and IL-8 (15). Therefore, we treated the selected cell types with the seven rIL-32 isoforms and measured the cytokine productions to evaluate the biological activity of each rIL-32 isoform and determine the dominant isoform in each cell type.

### Recombinant IL-32β, -γ, and -θ Induced Cytokines in Immune Cells

**Figure 4:**
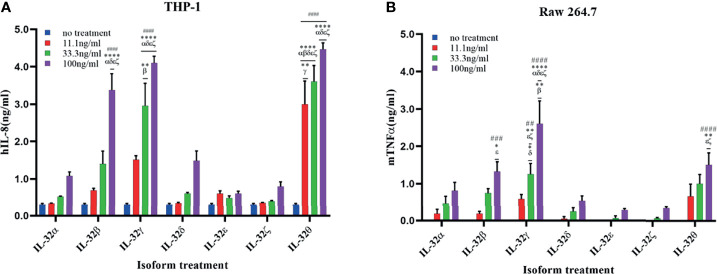
Biological activities of seven rIL-32 isoforms in immune cell lines. rIL-32 isoforms with different concentrations were treated in cells for 18 h. The levels of IL-8 and TNFα were measured in the supernatant of THP-1 **(A)** and Raw 264.7 **(B)**, respectively, by using ELISA. The bar graph represents the level of cytokines, mean ± SEM. Statistical testing was performed using two-way ANOVA followed by Tukey’s *post-hoc* analysis. ^##^ *p* < 0.01, ^###^ *p* < 0.001, ^####^ *p <*0.0001 as compared to no treatment control within the same isoform treatment. *p < 0.05, **p < 0.01, ****p < 0.0001 as compared to other displayed isoform symbols treated with the same concentration.

**Figure 5:**
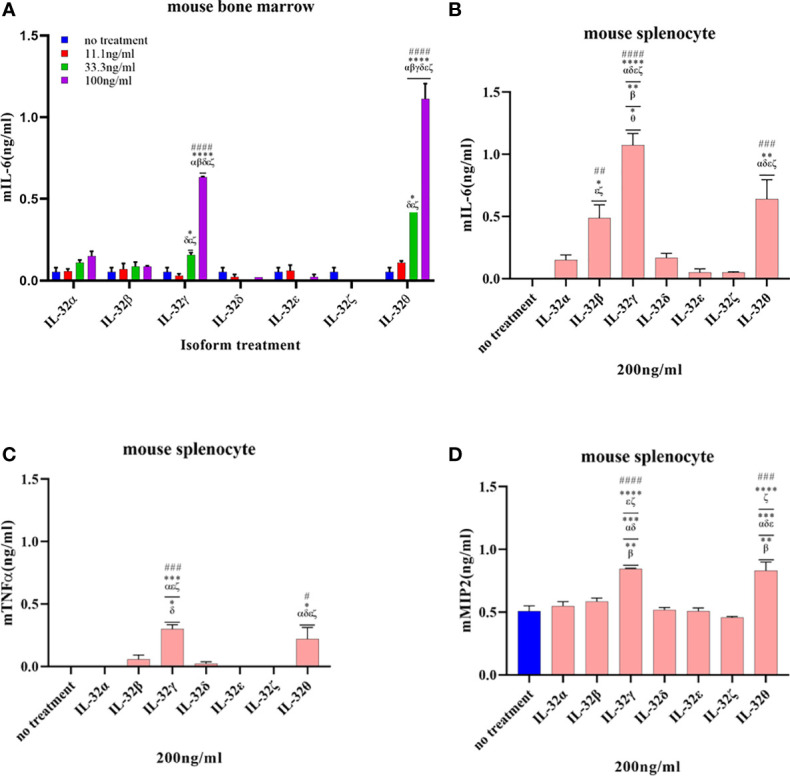
Biological activities of seven rIL-32 isoforms in the primary mouse immune cells. rIL-32 isoforms with different concentrations were treated for 18 h in mouse bone marrow. The level of IL-6 was measured by ELISA **(A)**, and 200 ng/ml of each isoform was treated for 18 h in mouse splenocytes. The levels of IL-6, TNFα, and MIP2 were measured by ELISA [**(B–D)**, respectively]. The bar graph represents the level of cytokines, mean ± SEM. Statistical testing was performed using two-way ANOVA **(A)** and one-way ANOVA **(B–D)**, followed by Tukey’s *post-hoc* analysis. ^#^ *p* < 0.05, ^##^ *p* < 0.01, ^###^ *p* < 0.001, ^####^ *p < *0.0001 as compared to the no-treatment control within the same isoform treatment. *p < 0.05, **p < 0.01, ***p < 0.001, ****p < 0.0001 as compared to other displayed isoform symbols treated with the same concentration.

The biological activity of the seven purified rIL-32 isoforms was assessed in immune cells. First, THP-1 and Raw 264.7 were stimulated with the seven rIL-32 isoforms, and a cell culture supernatant was used to assess the levels of IL-8 and TNFα production, respectively (**Figure 4**). Moreover, mouse isolated primary bone marrow and splenocytes were stimulated with the seven rIL-32 isoforms. Next, IL-6 from bone marrow as well as IL-6, TNFα, and MIP2 from splenocytes were assessed (**Figure 5**). The production of the measured cytokines was significantly increased by three rIL-32 isoforms, which were rIL-32θ, -γ, and -β isoforms, in a dose-dependent manner. These results were consistent in immune cell lines (THP-1 and Raw 264.7) and primary mouse immune cells (splenocytes and bone marrow cells). At the same time, the remaining four (rIL-32-α, -δ, -ϵ, and -ζ) isoforms have a weak or no activity in cytokine production.

In the case of THP-1 cells that were stimulated with different concentrations of rIL-32θ, these showed ±4 folds of IL-8 production compared to non-stimulated cells, and all three concentrations (11.1, 33.3, and 100 ng/ml) were significantly increasing the IL-8 levels, followed by rIL-32γ with concentrations of 33.3 and 100 ng/ml and then rIL-32β with a higher concentration only 100 ng/ml, which thus significantly induced IL-8 production. Interestingly, only rIL-32θ induced a significant amount of IL-8 production at a low concentration. Thus, rIL-32θ was considered the most potent rIL-32 isoform in this cell line. On the other hand, both rIL-32α and rIL-32δ showed ±1 ng/ml production of IL-8 at their highest concentration of 100 ng/ml. rIL-32ϵ and rIL-32ζ did not induce IL-8 production (**Figure 4A**).

The results from Raw 264.7 cells were similar to the THP-1 results, with one difference in the dominant isoform, which was rIL-32γ showing ±2.5 folds of TNFα production instead compared to non-stimulated cells at the concentrations of 33.3 and 100 ng/ml. However, rIL-32θ and rIL-32β induced significant TNFα production at 100 ng/ml (**Figure 4B**).

Next, the seven rIL-32 isoforms were treated in primary mouse bone marrow cells and splenocytes. The effect of each rIL-32 isoform on the production of cytokines is shown in **Figure 5**. In bone marrow cells, all rIL-32 isoforms at a low concentration (11.1 ng/ml) did not induce IL-6 production. Only two isoforms (rIL-32γ and rIL-32θ) induced the production of IL-6 at 33.3 and 100 ng/ml. However, the levels of IL-6 were significantly higher at 100 ng/ml of rIL-32θ, followed by rIL-32γ isoform (**Figure 5A**). Concurrently, rIL-32γ and rIL-32θ imply significantly higher IL-6, TNFα, and MIP2 in the primary mouse splenocytes as shown in **Figures 5B**–**D**, respectively.

### Recombinant IL-32α, -β, -δ, and -θ Induced Cytokines in Lung Cells

**Figure 6:**
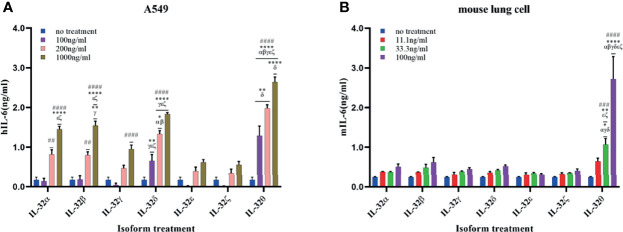
Biological activities of seven rIL-32 isoforms in lung cells. rIL-32 isoforms with different concentrations were treated for 18 h in human A549 lung cells **(A)** and primary mouse lung cells **(B)**. IL-6 production was measured by ELISA. The bar graph represents the level of cytokines, mean ± SEM. Statistical testing was performed using two-way ANOVA, followed by Tukey’s *post-hoc* analysis. ^##^ *p* < 0.01, ^###^ *p* < 0.001, ^####^ *p* < 0.0001 as compared to no-treatment control within the same isoform treatment. *p < 0.05, **p < 0.01, ****p < 0.0001 as compared to other displayed isoform symbols treated with the same concentration.

Biological activity was assessed in human A549 lung cells and primary mouse lung cells. Both cells were treated with rIL-32 seven isoforms of different concentrations for 18 h; then, the levels of IL-6 were assessed (**Figure 6**). In both lung cells, rIL-32θ showed a highly significant production of IL-6 than the other six isoforms in a concentration-dependent manner. However, unlike immune cells, rIL-32γ showed weak or no biological activities in A549 (**Figure 6A**) and mouse isolated lung cells (**Figure 6B**), respectively. Moreover, rIL-32δ, -β, and -α demonstrated a significant biological activity at high concentrations, in terms of IL-6 production, only on A549 cells. The remaining isoforms, rIL-32ϵ and rIL-32ζ, still have a very weak activity on A549 cells.

### Recombinant IL-32β, -δ, and -θ Induced Cytokines in Fibroblast Cells

**Figure 7:**
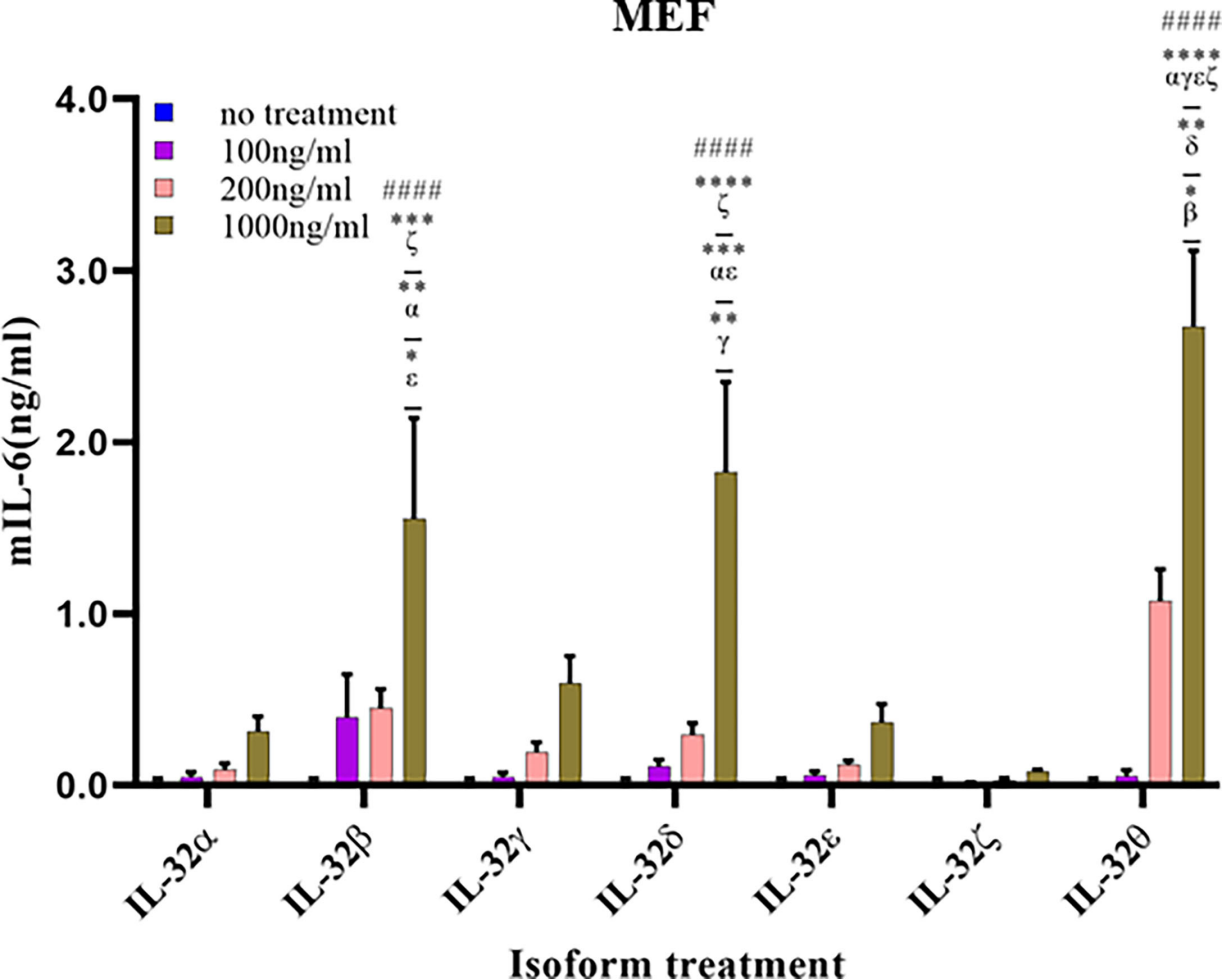
Biological activities of seven rIL-32 isoforms in mouse embryonic fibroblast. rIL-32 isoforms with different concentrations were treated for 18 h. The bar graph represents the level of IL-6 production, mean ± SEM, measured by ELISA. Statistical testing was performed using two-way ANOVA, followed by Tukey’s *post-hoc* analysis. ^####^ *p* < 0.0001 as compared to no-treatment control within the same isoform treatment. *p < 0.05, **p < 0.01, ***p < 0.001, ****p < 0.0001 as compared to other displayed isoform symbols treated with the same concentration.

Fibroblasts are cells that are mainly accountable for maintaining the extracellular matrix and are found within many tissues and organs such as the skin and lungs. Therefore, we measured the production of IL-6 in MEF cells treated with different concentrations of the seven isoforms to assess their biological activity (**Figure 7**). Like the immune and lung cells, rIL-32θ showed the highest production of IL-6. Nevertheless, a significant induction was found only with the high concentration of isoform at 1,000 ng/ml. Moreover, IL-32δ and IL-32β also showed a significant production of IL-6 following rIL-32θ at the high concentration of 1,000 ng/ml. rIL-32α and rIL-32γ showed a slightly non-significant production of IL-6, whereas rIL-32ϵ and rIL-32ζ isoforms did not induce cytokine production.

## Discussion

IL-32 is a novel multifunctional cytokine involved in various cell functions, differentiation, pro- or anti-inflammatory cytokines stimulation, and apoptosis (15–22). This cytokine promotes the induction of crucial inflammatory cytokines such as IL-1β, TNFα, IL-6, IL-8, and MIP2 (1, 2, 15, 23). Its expression engages numerous pathogenesis disorders, including inflammatory, autoimmune diseases, cancers, and infections (6, 24–26). IL-32 is found to come up with different splice variants (7, 27). However, there are limitations on IL-32 isoform characterization and correlation to define biological processes or disease conditions.

In this study, we were able to purify seven rIL-32 isoforms and evaluate their biological activity in different cell types, which may shed light on the specific activities of these seven IL-32 isoforms. Among them, four rIL-32 isoforms (IL-32α, -β, -γ, and -δ) were previously purified (8). Moreover, the remaining three rIL-32 isoforms (IL-32ϵ, -ζ, and -θ) were successfully constructed and purified for the first time in this study.

The expression of IL-32 in normal tissue revealed a high expression among various cell types, *e*.*g*., lung cells, fibroblasts, and immune cells, including monocytes and bone marrow (**Figure 3**). IL-32 is highly associated as well with disease conditions relating to these cell types, like rheumatoid arthritis, COPD, asthma, atopic dermatitis (AD), and certain cancers (6, 10, 12, 24, 28, 29). Therefore, we investigated the differences in the biological activity of the seven IL-32 isoforms within immune cells, lung cells, and fibroblasts. This study illustrated the need for a fundamental activity study regarding each IL-32 isoform.

So far, the expression of IL-32 has been correlated with numerous autoimmune diseases, among them RA and IBD that were the most-studied conditions in this regard. In the case of RA and compared to both healthy controls and patients with osteoarthritis, IL-32 expression was higher in RA patients (30); moreover, the synovial biopsies of RA patients exhibit a reduction of IL-32 upon anti-TNFα treatment. This interchange between IL-32 and TNFα suggests an intensification of inflammatory processes in RA (18). Regarding IBD, IL-32 has been suggested to have a role in the pathogenesis of IBD as it promotes the production of TNFα, IL-6, and IL-1β cytokines (31). To a lesser extent, patients with autoimmune diseases, including psoriasis, granulomatosis with polyangiitis, myasthenia graves, and type 2 diabetes, have also demonstrated a higher serum level of IL-32 than healthy controls (32–34). This difference was linked to disorder severity, suggesting its usefulness in being an inflammatory marker and outcome predictor.

More recently, IL-32 is also involved in type 1 diabetes; its mRNA levels in beta-cells were higher than in those in control subjects (35). These results are in line with the outcome of Jhun et al., who found that IL-32, specifically the gamma isoform, hastens streptozotocin-induced type 1 diabetes (36). In addition to autoimmune diseases, IL-32 is involved in respiratory inflammation conditions, such as COPD and asthma (12–14, 29, 37, 38). Its expression in lung tissue is enhanced in COPD patients and was associated with the obstruction degree of airflow *in vivo* (12). Besides this, IL-32 was found to play a role in gastric inflammation and cancer (39, 40), altogether signifying the execution of IL-32 in several inflammatory conditions with different patterns that could be explained by the existence of different isoforms that play different roles. Nevertheless, many of these studies fail to convey the IL-32 isoforms concerning disease conditions.

Lately, with increasing inconsistent reports regarding the role of IL-32, there is a large agreement that these different functions may relate to the different IL-32 isoforms. As mentioned earlier, most of the previous studies assessed the level of IL-32 with lack of specific isoform consideration. However, limited studies have demonstrated a few properties of some isoforms—for example, IL-32α has shown pro- and anti-inflammatory properties as it induces pro-inflammatory cytokine expression, thus suppressing its inflammatory role in the spinal cord. Besides this, the ability of IL-32α to promote the differentiation of osteoclast has been reported (17).

IL-32β also has both pro- and anti-inflammatory properties; it induces cytokine production of both IL-10 and TNFα in phorbol-12-myristate-13-acetate-stimulated cells, K562, and THP-1, respectively (1, 41, 42). In addition, this isoform also improves the adhesion ability of inflammatory cells to activate endothelial cells along with the consequent induction of proinflammatory cytokines. Therefore, it is involved in vascular inflammation propagation and the modulation of lipid accumulation (43, 44).

The longest isoform, IL-32γ, exhibits mainly a pro-inflammatory property and accordingly induces pro-inflammatory cytokine expression. Moreover, IL-32γ promotes the migration of activated T cells *via* chemokine (C-C motif) ligand 5 (CCL5) production in dendritic cells (DCs), stimulates the maturation and activation of DCs, and therefore increases the production of IL-12 and IL-6 (45, 46). In ankylosing spondylitis joint, IL-32γ plays an enhancement role in the differentiation of osteoblast (47). In RA patients, the level of IL-32γ was found to be upregulated significantly in both CD14^+^ monocytes and synovial membrane (16, 48). Therefore, it has been suggested that this isoform activates osteoclasts and, subsequently, tissue resorption. Furthermore, IL-32γ has shown a potent antiviral activity *versus* several viruses, specifically influenza A virus, vesicular stomatitis virus, herpes simplex virus 2, and human immunodeficiency virus (49–52).

IL-32δ is another isoform that generally demonstrates a proinflammatory property; it inhibits the production of IL-10. This inhibition occurs through the modulation of IL-32β; thus, this observation reveals that IL-32 is controlled by its isoforms (53). On the contrary, IL-32θ has mainly anti-inflammatory effects and has an inhibitory role on monocyte differentiation (41, 54). In patients with acute myeloid leukemia, IL-32θ regulates the production of TNFα negatively (55). In addition, IL-32θ negatively regulates CCL5 expression, an inflammatory chemokine secreted in several conditions such as viral infection and cancer, at both mRNA and protein levels. This data suggests the intracellular modulator role of IL-32θ under inflammation (56). Additionally, the isoform of IL-32θ has been found to suppress epithelial–mesenchymal transition, resulting in inhibition of invasion and migration of colon cancer cells under *in vitro* and *in vivo* assessments (57). Lastly, for IL-32ϵ, its transcript was elevated in the IBD mucosa, thus suggesting a protective activity (58). However, the present study showed that the IL-32θ isoform has the most prominent activity among the seven IL-32 isoforms.

There has been a widespread acceptance that IL-32γ is the most biologically active isoform (6, 8, 19, 26), as recent results suggest such interpretation. This conclusion is probably attributable to IL-32γ as it is the most-studied isoform. Here our data is in line with the interpretation regarding IL-32γ, specifically within the immune cells along with IL-32θ isoform. In more detail, we observed a higher activity of IL-32θ isoform in human-derived monocytes, THP-1, followed by the IL-32γ isoform (**Figure 4A**). However, in mouse-derived cells, IL-32γ exhibits maximum activity. Remarkably, IL-32β activity was directly following IL-32γ and IL-32θ among the tested immune cells (**Figures 4** and **5**). It is noteworthy that IL-32 switches between its isoforms under certain conditions were reported to reduce the inflammation as a safety control. This shift of transcripts has been indicated between IL-32γ and IL-32β isoforms (19). A similar shifting may be the case with IL-32θ to reduce its potent activity. More investigation is needed to confirm this suggestion and thus specify the key exon/domain/peptide signal responsible for the splicing change in both cases as well as examine the possibility of dimerization of IL-32β to reduce its section.

So far, few studies have been conducted on IL-32θ compared to the IL-32γ isoform. Interestingly, IL-32θ was the most active isoform in most cell types except in mouse Raw 264.7 and splenocyte (**Figures 4B** and **5B**). These *in vitro* results using a human-derived cell line (A549) showed four active isoforms with a difference in activity as reflected by the different levels of IL-6 production; these are IL-32θ, -δ, -β, and -α, in descending order. In comparison, *ex vivo* results using mouse-derived cells demonstrated a significant IL-6 production only with the IL-32θ isoform. In addition, IL-6 production in MEF showed that IL-32θ has the highest significant activity, followed by IL-32δ and IL-32β with comparable results. As mentioned above, IL-32θ has been suggested to play an intracellular modulatory role in breast cancer cells (56, 59). On the other hand, a study conducted on asthma patients showed a lower IL-32γ compared to healthy controls (37) as well as rIL-32γ that showed a negative regulatory effect in an asthma mouse model (14). However, this report did not consider the behavior of the IL-32θ isoform.

Inflammatory skin conditions have revealed the changes in levels of IL-32 with restricted and conflicting data regarding isoforms. A study comparing patients with asthma, psoriasis, and AD to healthy subjects found that IL-32 was higher in asthmatic and AD patients’ serum (60). They suggest that the release of IL-32 is mainly from apoptotic cells in both conditions, which is also in line with their *in vitro* results. Therefore, they declared the usefulness of using IL-32 serum levels in diagnosis to examine patients with AD or asthma. In addition, they mentioned the possibility of targeting IL-32 as a therapeutic purpose. There are some contradicting reports of Al-Shobaili et al., Meyer et al., and Lee et al. on the one hand, whereas Al-Shobaili et al. and Meyer et al. have found that the levels of IL-32 are increasing in psoriasis and AD, respectively (22, 61). Lee et al. reported that IL-32 exhibits a suppressor role for AD (60). Thus far, additional studies are needed to explain the role of these isoforms in different stages and different stimuli and their impact on each other.

In summary, we purified seven rIL-32 isoforms using the *E. coli* expression system and evaluated their biological activities using various cell types. Along with rIL-32γ, rIL-32θ revealed similar or higher activities in all tested cells. However, the behavior of IL-32 isoforms could be different at baseline and other conditions, as it may be influenced by many factors, such as different stimuli, health/disease conditions, cell type, and genetic background. Moreover, rIL-32ϵ and rIL-32ζ both showed little or no activity in the tested cells. Nevertheless, our results indicated the necessity to illuminate each rIL-32 isoform. Therefore, both mRNA and protein levels, in the forthcoming studies, should be considered. Furthermore, specific monoclonal antibodies that recognize each isoform are needed to accomplish this need, such as in the case of IL-32γ (62).

## Data Availability Statement

The raw data supporting the conclusions of this article will be made available by the authors without undue reservation.

## Ethics Statement

All animal procedures were reviewed and approved by the Konkuk University Institutional Animal Care Committee.

## Author Contributions

SS and SL designed the study, analyzed the data, and performed the experiments. SS, SL, SK, TTN, AT, and JH, performed the experiments. HJ, YL, SCY, and Y-GK analyzed the data. Funding acquisition was carried out by HJ, H-YP, S-YK, Y-GK, and SK. SS and YH examined the data. AT edited the manuscript. SK designed the study, supervised the project, and wrote the manuscript. All authors contributed to the article and approved the submitted version.

## Funding

This paper was written as part of Konkuk University’s research support program for its faculty on sabbatical leave in 2022. This work was supported by the National Research Foundation of Korea (NRF-2021R1F1A1057397). This research was supported by the Main Research Program (E0210602-02) of the Korea Food Research Institute (KFRI), funded by the Ministry of Science and ICT. S-YK and Y-GK were supported by NRF-2021M3A9G1026605. SL was supported by NRF-2019R1I1A1A01057699.

## Conflict of Interest

The authors declare that the research was conducted in the absence of any commercial or financial relationships that could be construed as a potential conflict of interest.

## Publisher’s Note

All claims expressed in this article are solely those of the authors and do not necessarily represent those of their affiliated organizations, or those of the publisher, the editors and the reviewers. Any product that may be evaluated in this article, or claim that may be made by its manufacturer, is not guaranteed or endorsed by the publisher.

## Abbreviations

AD, atopic dermatitis; CCL, chemokine (C-C motif) ligand; COPD, chronic obstructive pulmonary disease; DC, dendritic cells; *E. coli*, *Escherichia coli*; IBD, inflammatory bowel disease; IL, interleukin; MIP2, macrophage inflammatory protein-2; MEFs, mouse embryonic fibroblasts; NK4, natural killer cell transcript 4; PR3, proteinase 3; rIL-32, recombinant interleukin-32; RA, rheumatoid arthritis; TNFα, tumor necrosis factor-α.
